# Photoreceptor Cell Death, Proliferation and Formation of Hybrid Rod/S-Cone Photoreceptors in the Degenerating *STK38L* Mutant Retina

**DOI:** 10.1371/journal.pone.0024074

**Published:** 2011-09-30

**Authors:** Ágnes I. Berta, Kathleen Boesze-Battaglia, Sem Genini, Orly Goldstein, Paul J. O'Brien, Ágoston Szél, Gregory M. Acland, William A. Beltran, Gustavo D. Aguirre

**Affiliations:** 1 School of Veterinary Medicine, University of Pennsylvania, Philadelphia, Pennsylvanis, United States of America; 2 Department of Human Morphology and Developmental Biology, Semmelweis University of Medicine, Budapest, Hungary; 3 School of Dental Medicine, University of Pennsylvania, Philadelphia, Pennsylvania, United States of America; 4 J.A. Baker Institute, College of Veterinary Medicine, Cornell University, Ithaca New York, United States of America; 5 Laboratory of Retinal Cell and Molecular Biology, National Eye Institute, National Institutes of Health, Bethesda, Maryland, United States of America; Universidade Federal do Rio de Janeiro, Brazil

## Abstract

A homozygous mutation in *STK38L* in dogs impairs the late phase of photoreceptor development, and is followed by photoreceptor cell death (TUNEL) and proliferation (PCNA, PHH3) events that occur independently in different cells between 7–14 weeks of age. During this period, the outer nuclear layer (ONL) cell number is unchanged. The dividing cells are of photoreceptor origin, have rod opsin labeling, and do not label with markers specific for macrophages/microglia (CD18) or Müller cells (glutamine synthetase, PAX6). Nestin labeling is absent from the ONL although it labels the peripheral retina and ciliary marginal zone equally in normals and mutants. Cell proliferation is associated with increased *cyclin A1* and *LATS1* mRNA expression, but CRX protein expression is unchanged. Coincident with photoreceptor proliferation is a change in the photoreceptor population. Prior to cell death the photoreceptor mosaic is composed of L/M- and S-cones, and rods. After proliferation, both cone types remain, but the majority of rods are now hybrid photoreceptors that express rod opsin and, to a lesser extent, cone S-opsin, and lack NR2E3 expression. The hybrid photoreceptors renew their outer segments diffusely, a characteristic of cones. The results indicate the capacity for terminally differentiated, albeit mutant, photoreceptors to divide with mutations in this novel retinal degeneration gene.

## Introduction

Mutations in the large repertoire of photoreceptor-specific or enriched genes are causally associated with inherited retinal diseases in both humans (RetNet: http://www.sph.uth.tmc.edu/RetNet/) and animals [1], [2]. While the involved genes vary, apoptotic cell death is the final common pathway in retinal diseases [3]. This results from activation of one or several cell death pathways that appear to be mutation/model specific, and results in degeneration and death of photoreceptors with eventual blindness [4], [5]. As photoreceptors are terminally differentiated, there is no compensatory neurogenesis to replace the dying cells.

Early retinal degeneration (*erd*) is an autosomal recessive canine retinal disorder caused by a mutation in *STK38L*, a novel serine-threonine kinase gene also known as nodal-related protein 2 (*NDR2*) [6], [7]. In *erd*, an exonic SINE insertion eliminates part of the N-terminal regulatory region that is conserved in the nuclear Dbf2-related (NDR) subclass of AGC protein kinases [8]. These kinases, NDR1, STK38L, LATS1 and LATS2, are involved in the control cell division and morphogenesis in various cell types, including neurons [8], [9]. *LATS1* and *STK38L* are expressed in retina, and may function as tumor suppressor (LATS1), or in the control of cell death and proliferation [7], [8], [10].

The disease is characterized by abnormal retinal function whereby the b-wave of the electroretinogram (ERG) fails to develop, and the ERG remains a-wave dominated, an indication of abnormal synaptic transmission to second order neurons in the ONL [6]. We now report that after photoreceptor differentiation is completed in *erd* there is a period of sustained photoreceptor proliferation and cell death that occurs independently in different cells, and the newly generated photoreceptors are hybrid rod/S-cones. These results demonstrate that terminally differentiated photoreceptors are able to proliferate and differentiate under the appropriate stimulus, and suggest a possible role for *STK38L* in the control of retinal cell division.

## Results

### Early rod defects, and rod opsin delocalization in *erd*

**Figure 1:**
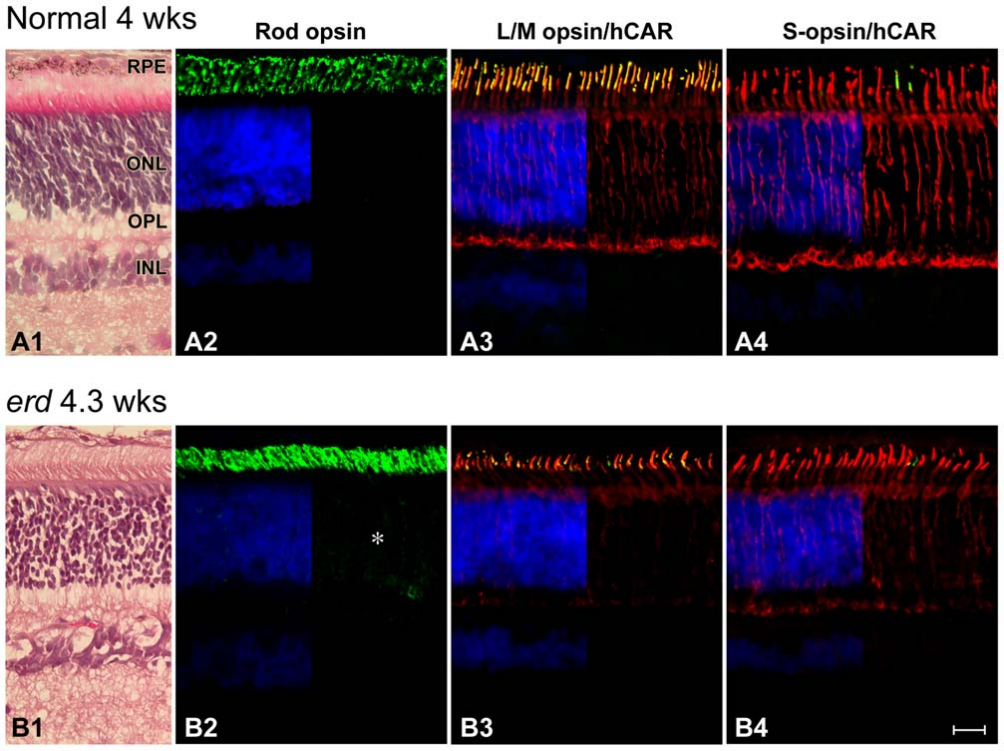
Normal expression of rod and cone molecular markers in early development in *erd*. Sections from 4 wk normal (*A*) and 4.3 wk mutant (*B*) stained with H&E (A1, B1), or labeled with antibodies against rod opsin (A2, B2; green), L/M-cone opsin (COS-1, green)/hCAR (red) (A3, B3), and S-cone opsin (OS-2, green)/hCAR (red) (A4, B4) with a DAPI (blue) nuclear stain. With the exception of rod opsin which shows slight delocalization of label into the outer nuclear (*) and plexiform layers, opsin labeling is restricted to the outer segments. hCAR labeling of cones is present throughout the cell. RPE = retinal pigment epithelium, ONL = outer nuclear layer, OPL = outer plexiform layer, INL = inner nuclear layer. Scale bar 40 µm.

**Figure 2:**
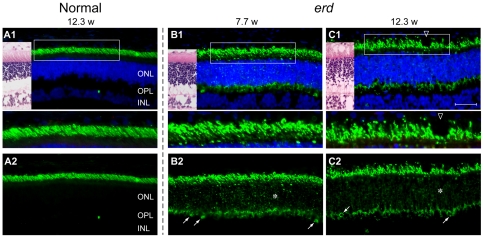
Early rod abnormalities in *erd*. Immunofluorescence labeling with anti-rod opsin antibody (green) shown with (A1, B1, C1) and without (A2, B2, C2) the blue DAPI nuclear stain in normal (A1, A2) and *erd*-mutant (B1, B2, C1, C2) retinas of different ages (weeks = w). *(A1, B1, C1)* Images of H&E stained adjacent cryosections are included for illustration. The boxed areas in the first row are presented at higher magnification in the panels below. *(A1, A2)* In normals, rod opsin labeling is restricted to the well oriented outer segments. *(B1, B2, C1, C2)* Rod outer segments are variable in length and irregular in *erd*, and opsin delocalizes to the inner segments, ONL (*) and OPL synaptic terminals (oblique arrows). The rod opsin delocalization is visualized best without DAPI. ONL = outer nuclear layer, OPL = outer plexiform layer, INL = inner nuclear layer. Scale bar 40 µm for principal panels.

In mutants, the early stages of retinal development were normal (Fig. 1A1, B1). Thereafter, rod outer segment (OS) shortening and loss was evident by 12.3 wks even though the outer nuclear layer (ONL) remained unchanged (Fig. 2A1,B1,C1). Rod opsin immunolabeling showed distinct abnormalities; OS were irregular and variable in length, and some cells showed opsin mislocalization to the ONL. These changes were present but mild at 4.3 wks (Fig. 1A2,B2), yet more marked later when there was extensive rod opsin mislocalization to the ONL and synaptic terminals, and rod opsin positive neurites sprouted into the inner nuclear layer (Fig. 2B1B2,C1C2; * and oblique arrows).

### Concurrent photoreceptor cell death or proliferation in developed mutant retina

**Figure 3:**
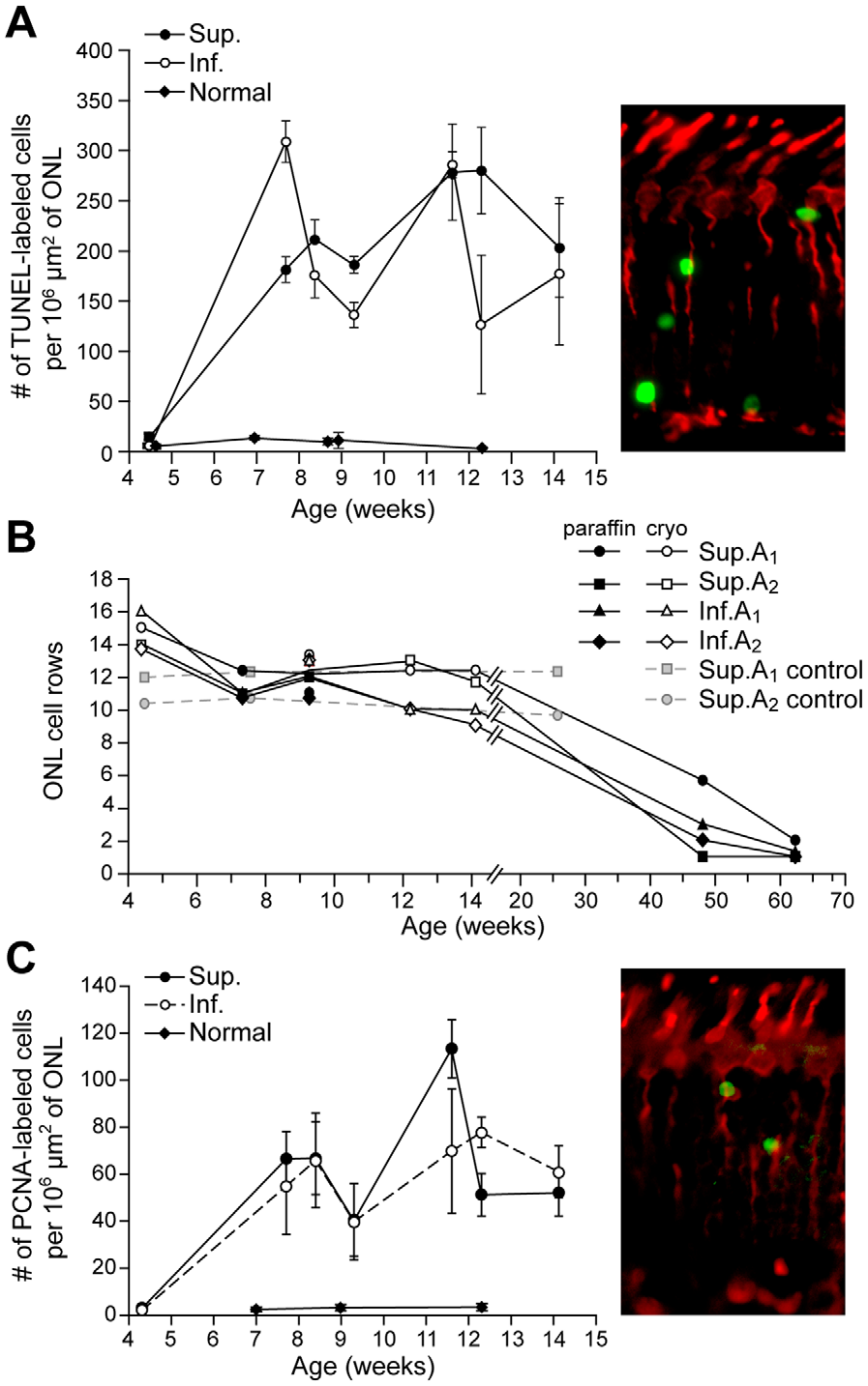
Photoreceptor cell death and proliferation in *erd*. (*A, C*) TUNEL and PCNA labeling, respectively, in *erd* is sustained between 7.7–14.1 wks with many labeled cells in the ONL of the superior (Sup.) and inferior (Inf.) meridians (data from both quadrants combined for normal; data points expressed as mean ±1 SD). Color insets illustrate the labeled cells (green) from a 7.7 wk old mutant animal for the corresponding assay in sections with hCAR antibody that labels all cones (red). (*B*) The number of photoreceptors in the outer nuclear layer (ONL), expressed as the mean number of rows of nuclei, remains relatively constant until 14.1 wks, and then decreases. Sup.A1/Inf.A1 = superior/inferior area 1, 2000±500 µm from the optic disc; Sup.A2/Inf.A2 = superior/inferior area 2, mid point ±500 µm between optic disc and ora serrata. For the ONL, the mean nuclei counts are presented. Between 4.3–14.1 wks of age, all the SD for ONL were ≤15% of the mean, with greater than 60% of the values being less than 10% of the mean.

TUNEL labeling was used to examine the kinetics of photoreceptor apoptosis/cell death in the disease during (4.3 wks), or after (7.7 wks) the completion of postnatal retinal differentiation [6]. As this could only be done in paraformaldehyde-fixed cryosections, analysis was limited to 4.3–14.1 wks. In 4 wk old normal and mutant, fewer than 13 TUNEL labeled cells/10^6^ µm^2^ of ONL were present (Fig. 3A), values similar to those reported in a previous study of normal dogs [11]. However, in mutants the number of TUNEL positive cells markedly increased thereafter, and labeled nuclei were distributed uniformly throughout the ONL, including the outermost level where cone somatas were located; similar numbers of TUNEL positive cells were found in central, equatorial and peripheral regions. The high number of apoptotic cells indicated that there was active and sustained cell death occurring in the 7.7–14.1 wk time period that was limited to the ONL.

Despite the ongoing photoreceptor cell death demonstrated by TUNEL labeling, there was no apparent loss of ONL cells (Fig. 3B). The ONL thickness in mutant retinas was constant and equal to that in controls until 14.1 wks of age. Thereafter the ONL decreased dramatically by 48.1 wks, and became even thinner at 62 wks.

To examine why ONL thickness did not change in spite of the high rate of photoreceptor apoptosis, we determined whether cell proliferation was occurring using an antibody directed against proliferative cell nuclear antigen (PCNA). Labeled cells were present in the mutant ONL during the period of sustained cell death, with values ranging from 40–113 PCNA labeled cells/10^6^ µm^2^ (Fig. 3C). The PCNA labeled nuclei in the ONL were morphologically similar to the TUNEL positive ones. To further characterize the cell proliferation findings in terms of cell location and retinal distribution, selected retinal sections from control (7 and 9 wks) and mutants (7.7 and 9.1 wks) were labeled with KI-67, another marker of cell division, and demonstrated a similar ONL labeling pattern as with PCNA (data not shown).

### Phospho-histone H3 (PHH3) labeling is limited to mutant photoreceptor cells

**Figure 4:**
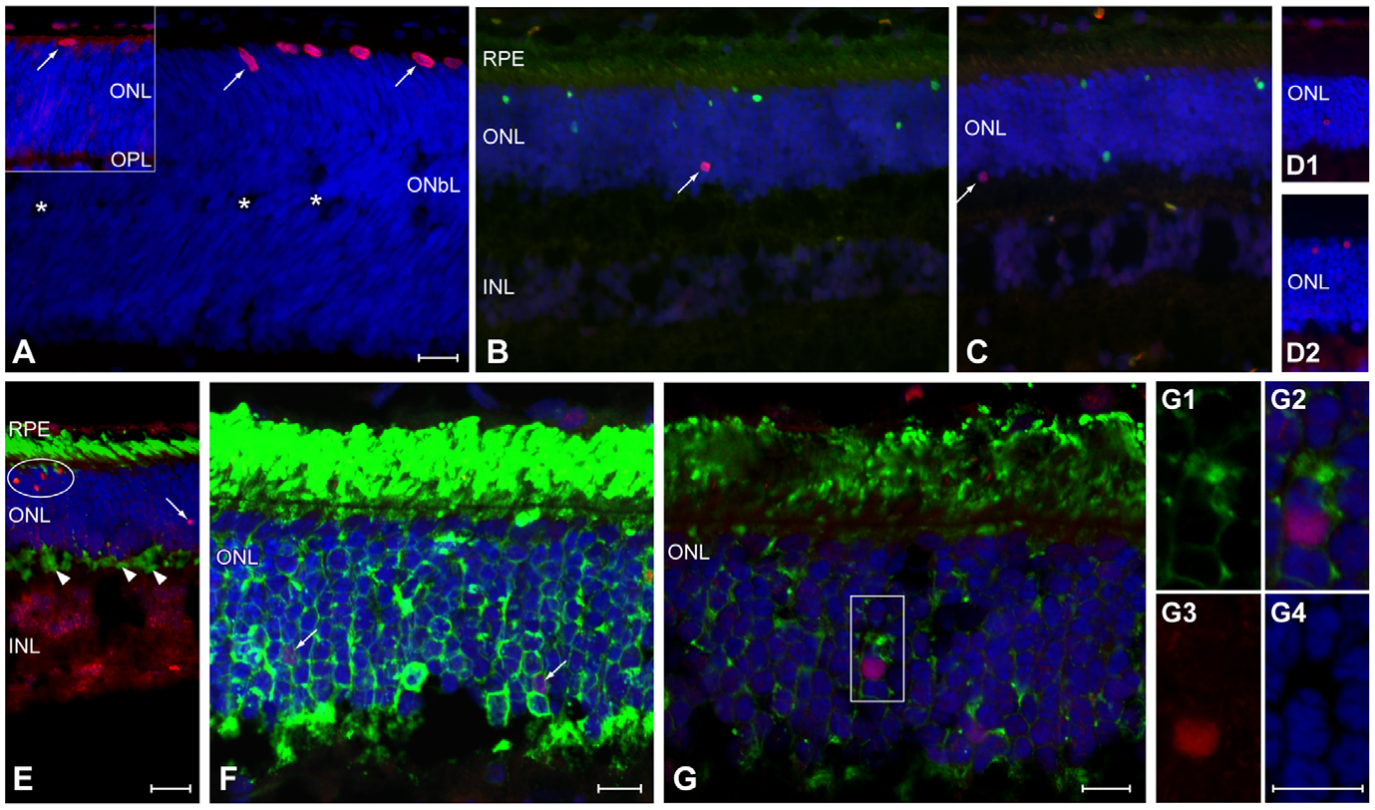
Phospho-histone H3 (PHH3) labeling of mitotic cells. Sections examined by epifluorescence (A–E) or confocal (F–G) microscopy using single (PHH3; A, D1, D2) or double labeling (B, C -TUNEL; E, F, G -rod opsin), and DAPI (blue) nuclear staining. (*A*) Cell division occurs in the normal retinal periphery of young dogs [1 and 1.6 (inset) weeks of age] as the outer neuroblastic layer (ONbL) begins to separate (*), and the outer and inner nuclear (ONL, INL) and outer plexiform (OPL) layers form. Labeled nuclei (arrows) are large, ovoid and located at the external edge of the retina. (*B, C, D1, D2*). In *erd*, PHH3 labeled nuclei are small, round (arrows), located at all levels of the ONL, and labeling does not co-localize with TUNEL labeled (green) nuclei (B,C and D1,D2 are different dogs at 11.6 wk of age). (*E,F*) Epifluorescence (E, 11.6 wks of age) and maximally projected confocal image (F, 7.7 wks of age) shows individual or clustered (arrows or oval, respectively) PHH3 labeled nuclei (orange or purple nuclei) in the ONL, and rod opsin (green) delocalization into the ONL and synaptic terminals (E, arrowheads). (*G, G1–G4*) Single confocal sections from a different region of the same retina as (F) using rod opsin (green), PHH3 (red-purple), and DAPI (blue) labeling. (*G1–G4*) Higher magnification of the boxed region showing colocalization of rod opsin labeling surrounding PHH3 (purple) labeled nucleus (G2), and the individual channels for rod opsin (G1), PHH3 (G3) and DAPI (G4). Scale bars: a–e = 20 µm, f–g, g1–4 = 10 µm.

PHH3 labeling was used to differentiate mitotic cells [12], [13] from those undergoing DNA repair that labeled with PCNA [14]. In normals, PHH3 labeled nuclei were located adjacent to the external limiting membrane, and, in the immediate postnatal period, limited to the retinal periphery at the time that the outer neuroblastic layer was separating, and the outer plexiform layer (OPL) had just formed (Fig. 4A, arrows). In control dogs 4 wks or older, there were almost no PHH3 labeled cells in ONL. In mutants, on the other hand, PHH3 labeled nuclei only were present at different levels of the ONL, and these were small, round and distributed uniformly from the center to the periphery; labeled cells were distinct from those undergoing apoptosis (Fig. 4B–E). Rod opsin and PHH3 labeling clearly demonstrated colocalization, and labeled nuclei were enclosed by a rod opsin positive cytoplasmic rim (Fig. 4F,G,G1–4).

We have examined a subset of samples and calculated the number of PHH3 positive cells using the same method used for counting PCNA positive cells (see Materials and Methods). The results indicate that comparable numbers of labeled nuclei in the ONL, expressed as labeled cells/10^6^ µm^2^, were present with both PCNA (7 wk control = 2±1; *erd*-7.7 wk = 61±16 and 11.6 wk = 91±30) and PHH3 (7 wk control = 4±2.3; *erd*-7.7 wk = 153±56 and 11.6 wk = 100±11) labeling.

### Müller cells, stem cells or microglia do not contribute to the population of dividing ONL cells

Double labeling with PHH3 and glutamine synthetase (GS) was used to rule out Müller cell contribution to the dividing cell population. Distinct and comparable GS labeling was present in the normal and mutant retinas, and extended from the external to the internal limiting membranes. In *erd*, GS labeling was not associated with PHH3 labeled nuclei in the ONL (Fig. S1). An antibody against nestin, expressed in neuronal stem cells, was used to label the normal and mutant retinas in the 4.3–14.1 wk time period. In both groups, comparable nestin labeling was found only in the retinal periphery and ciliary margin region, the site of retinal stem cells [15], but not in the ONL (Fig. S2A1, A2 illustrate the finding for 9 and 8.3 wk control and *erd*, respectively). Nestin, PCNA and PHH3 gave similar labeling results for the periphery/retinal ciliary margin region of normal and mutants (data not shown). Antibodies against CD18 or PAX6, respectively, labeled microglia (CD18) or Müller cells and inner retinal neurons (PAX6), and the results were comparable between *erd* and control (Fig. S2B1,B2 and C1,C2). Taken together, the results show that the PCNA or PHH3 labeled cells in the ONL could not be accounted for by dividing Müller cells, stem cells, microglia or macrophages responding to the degenerative events in the photoreceptor layer.

### Cones and formation of hybrid rod/S-cone photoreceptors

**Figure 5:**
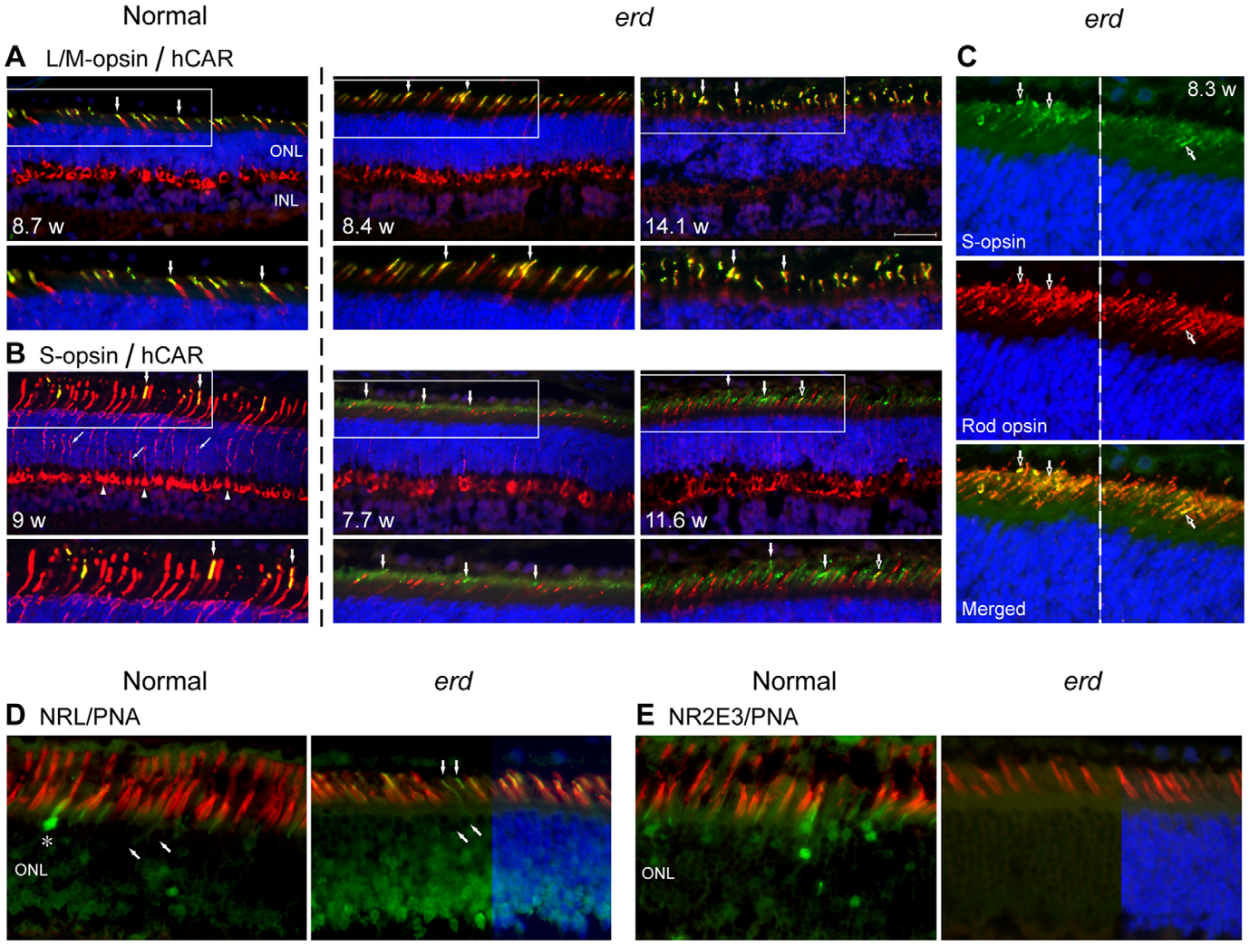
L/M-, S- cones, and hybrid rod/S-cones in the *erd* retina. Double immunofluorescence (A: L/M-opsin = green, hCAR = red; B: S-opsin = green, hCAR = red; C: S-opsin = green, rod opsin = red; D: NRL = green, PNA = red; E: NR2E3 = green, PNA = red; DAPI nuclear stain = blue) in normal and *erd* retinas of different ages (weeks = w). The boxed areas in A and B are presented at higher magnification in the panels below, and vertical arrows identify the same cells. (*A, B*) Control cones label distinctly but variably with hCAR; L/M-cones labeled with COS-1are more numerous, and only few S-cones are present. Other than cone outer segment disorientation in the older *erd* retina, L/M-cone opsin labeling is normal and restricted to the outer segments of these cells. In mutants, the OS-2 antibody distinctly labels S-cone outer segments, and also there is more diffuse but less intense labeling over the rod outer segments. (*C*) Colocalization of S-opsin and rod opsin labeling in hybrid rod/S-cone photoreceptors is demonstrated in single and merged images. Because the S-opsin (green) labeling is weaker than rod opsin (red) in rods, the green signal has been enhanced in the merged image to illustrate colocalization. Closed (A, B) or open (C) arrows identify the same cells. (*D, E*) Double labeling with PNA and NRL or NR2E3, respectively, in normal and mutant retinas. In the mutant, NRL labels the outer segments of presumable hybrid rod/S-cones (vertical arrows) that are not invested by a PNA positive insoluble cone extracellular matrix; oblique arrows point to cone nuclei which are not labeled and * identifies an NRL labeled cone nucleus in normal retina. The mutant retina shows absence of NR2E3 labeling. ONL = outer nuclear layer, INL = inner nuclear layer. Scale bar 40 µm for principal panels in A, B, and 20 µm for C–E.

Antibodies against hCAR and PNA were used to identify both cone classes, and evaluate their structure and distribution during development and degeneration. Control cones were uniformly elongated, and PNA labeled the insoluble matrix domain around OS. Labeling with hCAR also was distinct in all cone compartments, but variable in the axons coursing through the ONL (Fig. 1A3,A4, 5A,B). Mutant cones, on the other hand, appeared shorter in younger animals (Fig. 1B3,B4), and some failed to show hCAR labeling even though the cells were readily identifiable in transmitted light with or without DIC optics. Qualitative assessment of cone numbers based on PNA labeling was normal; however, immunolabeling with antibodies against cone opsins indicated that in the older mutants the OS were shorter and irregular (Fig. 5A,B).

The COS-1 and OS-2 antibodies, respectively, labeled the L/M- and S-cone classes comparably in control and mutants, and labeling was restricted to the OS (Fig. 1A3,A4, B3,B4, 5A,B) [16]. To determine qualitative immunolabeling intensity differences between normals and mutants, an antibody dilution series was carried out. The minimal dilution required for labeling S-cones was the same for control and mutants for the 3 antibodies (monoclonal OS-2 (1∶400), goat sc-14363 (1∶20,000), rabbit AB5407 (1∶40,000)). In contrast, polyclonal antibodies sc-22117 and AB5405 against the L/M-cones resulted in distinct labeling at dilutions of 1∶20,000 and 1∶80,000, respectively in *erd*, but required double that concentration (1;10,000 and 1∶40,000) for comparable labeling in normal cones.

An unexpected finding was the labeling of both S-cones and rod OS with the OS-2 antibody. This was observed in the 7.7–14.1 wk time period, but not at 4.3 wks where OS-2 labeling was restricted to S-cones, and similar to control (Figure 1A4,B4). At 7.7 wks, however, OS-2 labeling of rod OS was faint and homogeneous, but clearly localized to the photoreceptor OS layer. By 11.6–14.1 wks, labeling of rods was more intense (Figure 5B). Double labeling with OS-2 and rod opsin antibodies indicated that many of these rods showed colocalization of both proteins in the same OS (Figure 5C). In contrast, labeling with COS-1 or the two polyclonal red/green cone opsin antibodies indicated that label was restricted only to hCAR positive L/M-cones (Figure 5A).

To determine if rod OS labeling was dependent on antibody concentration, we carried out a qualitative immunolocalization comparison between control (7 wks) and mutant (7.7 and 11.6 wks) retinas using different OS-2 antibody dilutions. In controls, S-cone OS were intensely labeled at 1∶100 and 1∶200 dilutions, but weakly at 1∶400, and rods were unlabeled. The mutant retina showed labeling of rod or S-cone OS at 1∶100 and 1∶200, and the intensity, by photoreceptor class, was equal at each dilution. At 1∶400 only S-cone OS were labeled with an intensity comparable to lower dilutions.

As the transcription factors NRL and NR2E3 are involved in retinal cell fate specification, we examined the expression of these two gene products in normal and mutants at the time when hybrid rod/S-cones were present. Both show NRL expression in rod and cone inner segments (IS), cone OS and rod nuclei; as well *erd* retinas expressed NRL in presumptive hybrid rod/S-cones as these cells lack a PNA labeled domain (Fig. 5D). In contrast, NR2E3 is mainly expressed in nuclei of rods in normals, although occasional labeling of cone nuclei and IS also was observed. The mutant retina showed absence of NR2E3 labeling in the ONL or photoreceptor layer (Fig. 5D).

### Gene expression in normal and mutant retinas

**Figure 6:**
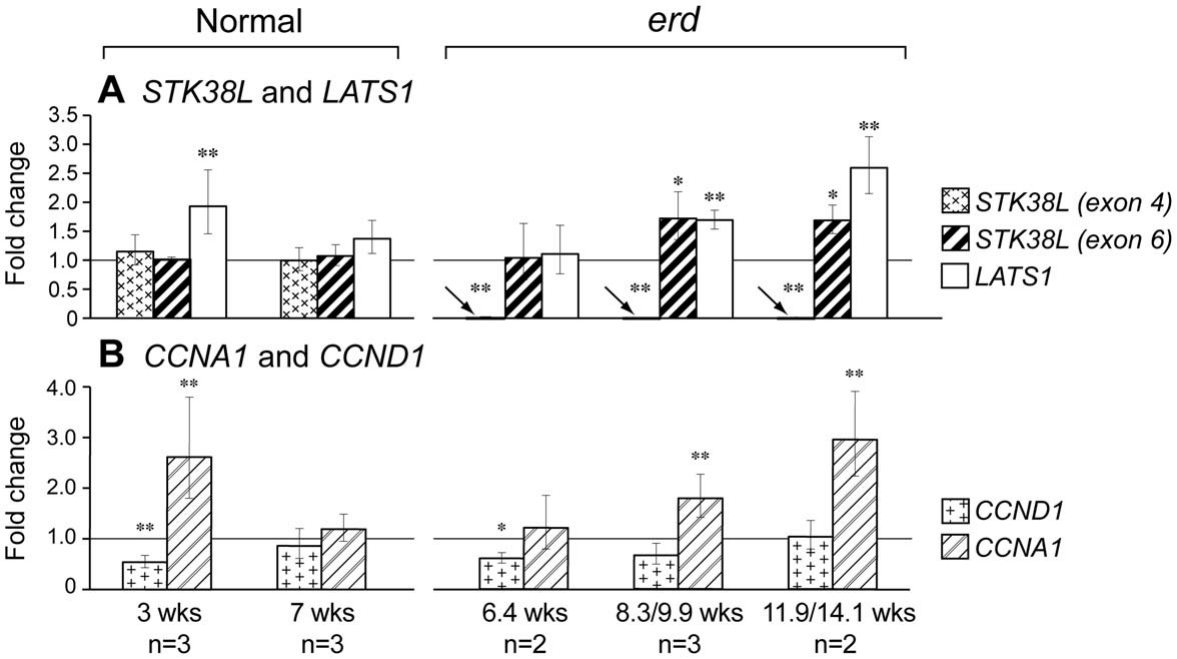
Retinal gene expression changes during normal development and disease. Expression was analyzed for genes that are (*A*) causally associated with the mutation or are part of the same gene family, or (*B*) involved with the cell cycle. Statistical significance between different groups in comparison to the 16 wks old normal control retinas (n = 3), depicted by horizontal line at 1.0 Fold Change, was assessed with an unpaired *t*-test, and expressed as statistically significant (** = p≤0.05) or towards statistical significant (* = 0.05≤p≤0.1). For *STK38L*, one probe was located within the exonic deletion [*STK38L (exon 4)*], and the second in exon 6, 3′ to the splicing defect [*STK38L (exon 6)*]. Noted below each time point are the number of different samples used (n = ) at each time point.

We used qRT-PCR to characterize retinal expression of the mutated gene, *STK38L*, and another member of the NDR family, *LATS1*, at different ages during normal development and disease (Fig. 6A). *STK38L* expression in control retinas was unchanged during development. Although mutant mRNA lacked exon 4, the altered transcript showed a slight increase in expression at the 2 older disease time periods examined when using an exon 6 probe (0.05≤p≤0.1). *LATS1* expression was increased at the 3 wk time point in normals, and at the 2 older disease time points. As well, we examined by qRT-PCR the expression pattern of two cell cycle genes. The expression pattern of*CCNA1* was similar to *LATS1*, but *CCND1* expression was minimally reduced or unchanged at the three disease stages examined (Fig. 6B).

**Figure 7:**
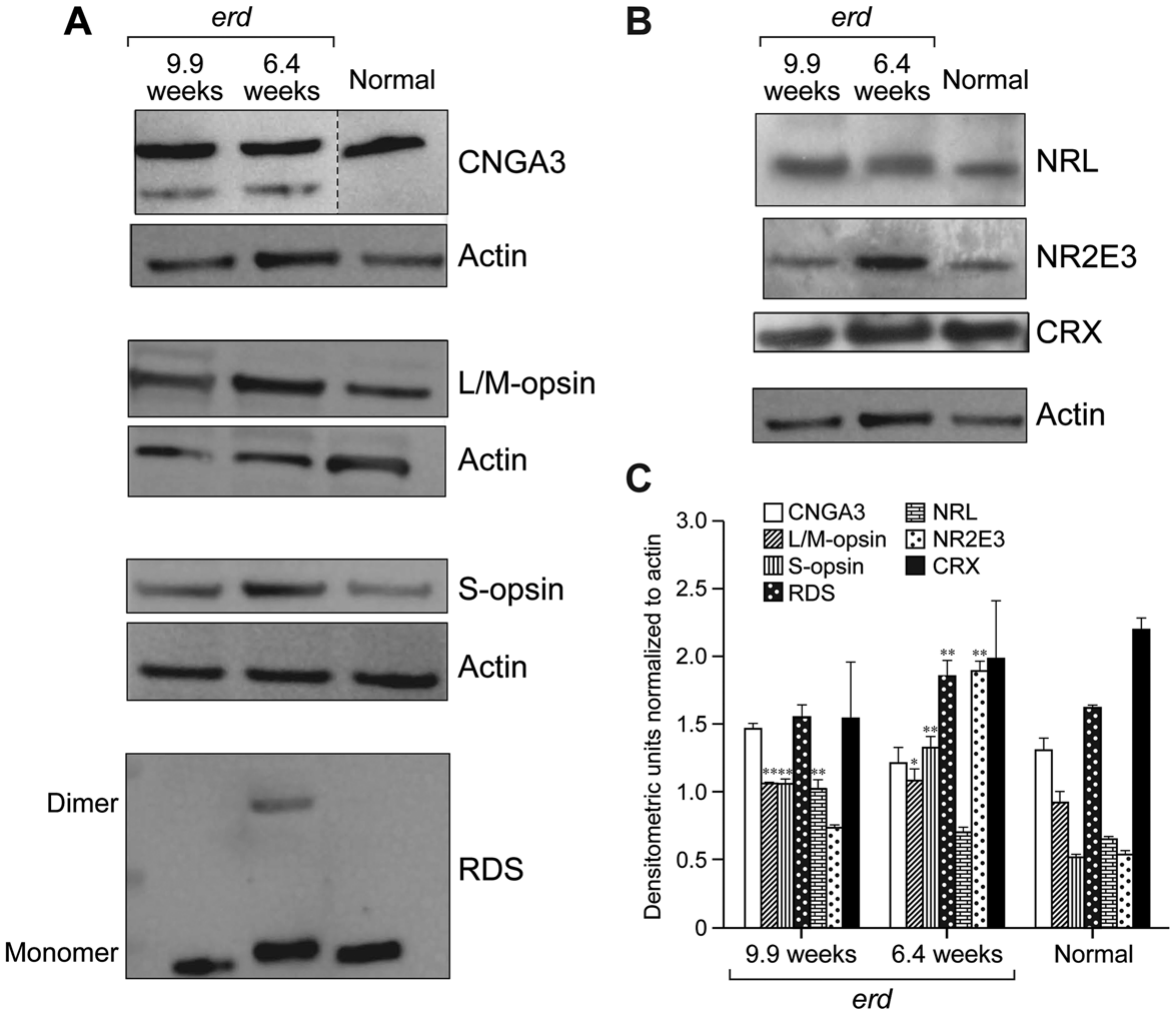
Protein expression in *erd* retina. Expression of (*A*) CNGA3, L/M-opsin, S-opsin, RDS, and (*B*) NRL, NR2E3, CRX proteins in cleared whole retinal lysates. (*C*) Net intensity densitometric values are expressed as corrected net intensity of the sample normalized to corrected net intensity of actin loading controls. Actin loading control is the same for S-opsin and RDS, and for NRL, NR2E3 and CRX, respectively. Results are the mean ± SD of four independent densitometer scans of two individual westerns. Confidence levels: * = 95%, ** = 99%.

Immunoblotting demonstrated increased expression of three of four photoreceptor-specific proteins evaluated (S-opsin, L/M-opsin and RDS peripherin) in lysates from 6.4 and 9.9 wk *erd*-retinas compared to 8 wk normal control (Figure 7A,C). The CRX transcription factor was unchanged, but NR2E3 was significantly increased at 6.4 wks, whereas NRL levels were elevated only at 9.9 wks (Figure 7B,C). The NR2E3 results differed from those obtained by immunocytochemistry, and could be the result of different antibodies used in the blots and tissue sections as well as accessibility of antibody to epitope in the sections.

### Cone-like outer segment renewal in *erd* mutant retina

**Figure 8:**
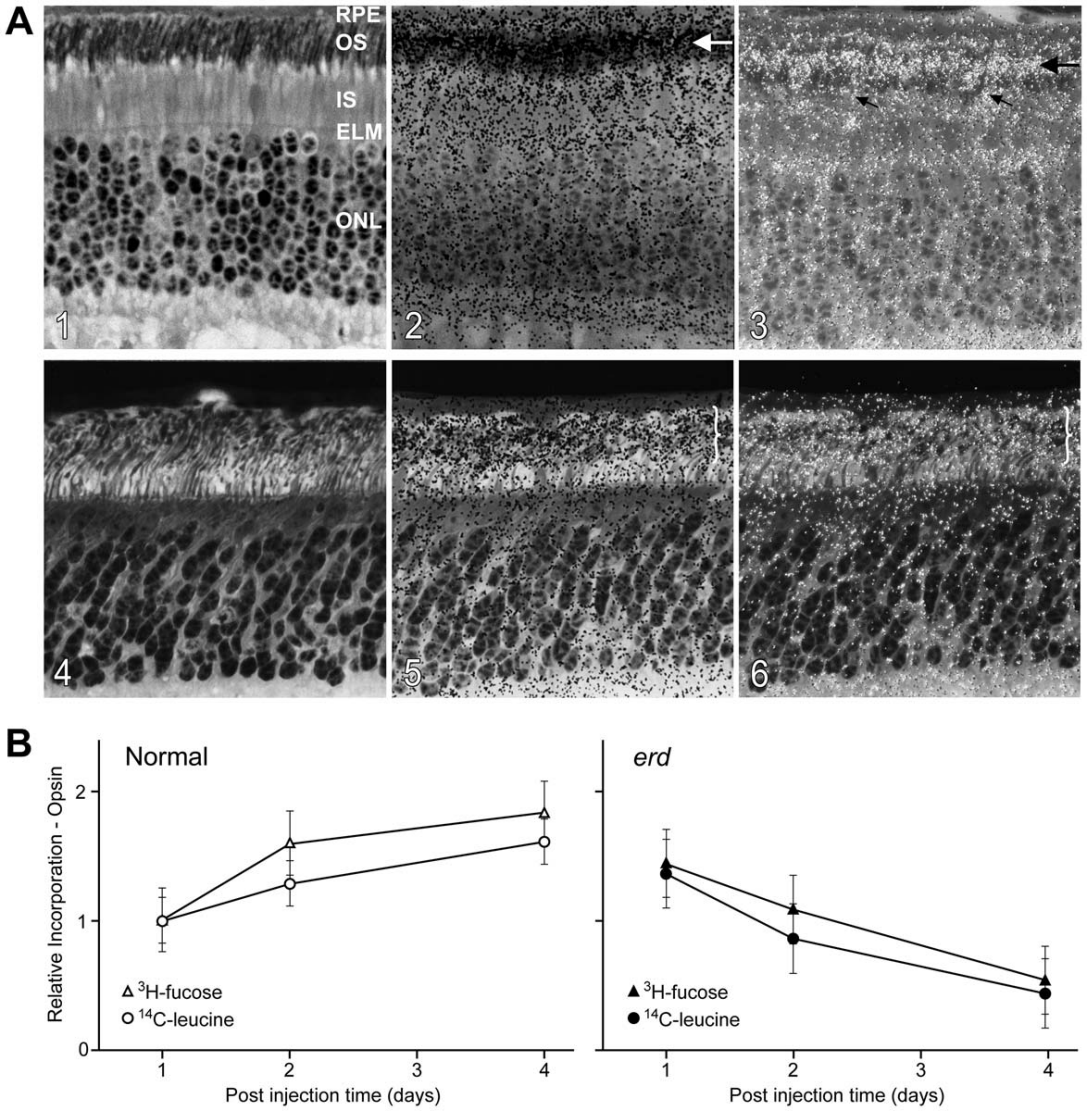
Abnormal outer segment renewal in *erd* rods. *(A1–3)* Rod outer segment renewal examined 4 days following intravitreal injection of ^3^H-fucose in 10.9 week old normal and *(A4–6) erd*-affected dogs. The images (A1,A2 and A4,A5) are serial sections taken from the posterior pole of the superior retinal quadrant. The transmitted bright-field autoradiogram (A2) shows in the normal a band of labeling whose leading edge extends to 2/3 of the rod OS length (A2, horizontal arrow) with a trailing tail of radioactivity. This is better visualized in the combined transmitted/epipolarizing image (A3, arrow) that also shows diffuse label in the cone OS (oblique arrows). The mutant retina (A4–6) shows diffuse label at all levels of the OS layer (A5,A6; brackets) that is similar in rods and cones. RPE = retinal pigment epithelium; OS = outer segments; IS = inner segments; ELM = external limiting membrane; ONL = outer nuclear layer. (*B*) Relative incorporation of ^3^H-fucose/^14^C-leucine into opsin at different time points following a single intravitreal injection. All data are normalized to incorporation of label at day 1. The *erd* outer segments initially take up both labels (day 1), but label intensity decreases at later post injection times, suggesting that label is diffusely distributed through the rod outer segment membranes after discs are formed, and outer segment tips are lost to the RPE by daily shedding/phagocytosis events.

Autoradiography of control retinas following intravitreal ^3^H-leucine or ^3^H-fucose injection showed renewal by band displacement in OS characteristic of rods (Figure 8A1–A3, horizontal arrows). Calculated renewal rates were 2.13±0.08 µm/day for central and mid peripheral regions. In mutants, a distinct band of radioactivity was not appreciable at any time, and a renewal rate could not be established (Figure 8A4–A6; } illustrates diffuse label). Diffuse label was present at all levels of the OS layer, and the labeling pattern was similar in rods and cones. At the 4 day post injection point, this difference clearly was apparent; in controls, the band of radioactivity, and a trailing label tail, was present distal to the rod OS midpoint, but mutants demonstrated diffuse label and no band.

### Normal rod opsin biosynthesis in mutant retina, but abnormal renewal kinetics

Relative incorporation of ^3^H-fucose/^14^C-leucine into normal and *erd* rod opsin differed (Figure 8B). In normals, opsin labeling with both precursors increased at post injection days 2 and 4 as the labeled precursor pool in the vitreous continued to be incorporated into newly synthesized protein. Regression analysis of combined ^3^H-fucose and ^14^C-leucine data from controls yielded a slope (daily increase in label) of 0.225±0.0746. In contrast, the mutant retinas demonstrated decreased labeling at 2 and 4 days. Regression analysis on the combined data yielded a slope of −0.296±0.0459, an indication of loss of radioactivity over the time period examined. The difference between control and affected slopes was highly significant (Z = 5.9401, p = 1.43E-09).

## Discussion

In the *STK38L* mutant retina, rods and cones begin to differentiate normally, and express polarized molecular markers, e.g. opsins and cone arrestin, indicating that cell class specification occurred after the final cell division [17]. Subsequently, rods show disparate lengths of the OS, neurite sprouting, and opsin mislocalization. These changes are soon followed by cell death primarily affecting rods and then cones. Apoptosis detected by TUNEL labeling occurs in terminally differentiated photoreceptors after retinal development is completed; it is sustained, and many dying cells are present throughout the ONL between 7.7–14.1 wks of age [6].

An unexpected finding, however, is that the high levels of TUNEL labeling is not accompanied by concomitant photoreceptor loss as is characteristic for inherited retinal degenerations, and the mutant ONL remains at a constant thickness until after 14.1 wks of age [5], [11]. This suggests that a concurrent, compensatory proliferative event must be occurring in mutants, and was confirmed by PCNA labeling. The antibody labeled many cells in the *erd* ONL, but almost none in controls. PHH3 and rod opsin double labeling confirmed that photoreceptor cells were undergoing mitosis rather than DNA repair as the number of labeled cells were comparable, and there was no co-localization of TUNEL and PHH3 labeling [13], [14]. Furthermore, as labeling with antibodies against CD18, GS, PAX6 and nestin ruled out the possibility that the ONL mitotic cells are microglia, Müller or stem cells, we conclude that the proliferating cells must be photoreceptors.

We have considered the possibility that the proliferating and dying cells are the same, and that lack of colocalization of TUNEL and PHH3 labeling results from the cells dying at a stage in the cell cycle when the proliferation marker is not expressed. This possibility, however, is not consistent with the results. If cell proliferation were occurring, and TUNEL labeling reflects only those cells that are replicating their DNA, we would have expected to see a marked increase in ONL thickness. We previously have observed such proliferative events in young dogs with an *RPGR* mutation following intravitreal CNTF administration [18].

Thus between 7.7 and 14.1 wks of age, mutant photoreceptors are undergoing apoptosis or cell division. As the ONL thickness remains unchanged during this time, the magnitude of both events has to be comparable, otherwise decreases or increases in ONL cell number would occur. Because rods are uniformly distributed throughout the ONL and outnumber cones 22∶1 outside the area centralis [19], it is likely that rods are the predominant cells that are dying or proliferating. The absence of TUNEL or PCNA labeled cells at the earlier time point suggests that terminally differentiated albeit mutant photoreceptors commit to the cell death or proliferation fate.

Neurogenesis in the developed mammalian retina or CNS is limited. In the brain, the subventricular zone of the lateral ventricle, and the dentate gyrus of the hippocampus show low level of neurogenesis from neuronal progenitors [20], [21]. In the retina, and under specific circumstances [22], a limited number of photoreceptors and other neurons are generated from presumably terminally differentiated Müller cells that dedifferentiate, proliferate and express neuronal progenitor markers; this has been reported in the adult rat, mice, chicken and fish (see [23] for review). In contrast, rod photoreceptor replacement in zebrafish is dependent on the extent of injury. Total rod ablation results in robust Müller cell proliferation and formation of neuronal progenitor clusters, while ablation of a subset of rods results in proliferation of rod precursors [24].

Other than the present study, generation of new photoreceptors in a naturally occurring retinal degeneration has not been reported. A previous study suggested the possibility of photoreceptor proliferation based on PCNA and BrdU labeling in *rd1* retinas [25]. However, specific labeling for microglia subsequently showed that all dividing cells in the ONL were microglia rather than photoreceptors [26].Thus the *STK38L* mutant retina appears to be unique in having terminally differentiated photoreceptors undergoing cell proliferation.

What is not clear at this time are the signals that commit terminally differentiated photoreceptors to die or divide. While apoptosis is the final common pathway in retinal degenerative diseases, there are multiple potential pathways that link the mutation to the apoptotic event, and these are yet to be defined for most species including dogs [3], [5]. In *erd*, cell proliferation likely results from loss of part of the N-terminal regulatory region that is highly conserved in all NDR subclass of AGC protein kinases [8]. The exonic SINE insertion removes exon 4 from the mature RNA, and eliminates the binding sites for S100B and Mob proteins, part of the protein kinase domain, and a Thr-75 residue critical for autophosphorylation [7]. We posit that in the normal retina, the terminally differentiated photoreceptors are kept from dividing by NDR2-Mob1 interaction. Removing this control in mutants allows the cell to re-enter the cell cycle and divide, as suggested by increased *cyclin A1* expression [8]. Increased expression of *LATS1* could be an attempt to suppress this proliferation. Photoreceptor cell division, however, is temporally limited as the ONL thins after 14.1 wks. The cell division events are similar to what has been observed with the transgenic expression of SV40 large tumor antigen in mouse retina that results in postnatal DNA synthesis and mitosis. A major difference from *erd*, however, is that after mitosis, the transgenic photoreceptors do not progress to a viable postmitotic stage and die [27].

Coincident with the photoreceptor cell death and proliferation phase is a change in the visual cell population from the preexisting normal mosaic of L/M- and S-cones, and rods. Although both cone types remain, most rod cells that presumably were generated after proliferation now become hybrid photoreceptors that express both rod opsin and cone S-opsin. This is supported by absent NR2E3 expression in mutant photoreceptors, at least by immunocytochemistry, resulting in rod-like photoreceptors that express cone genes [28], [29]. The discrepancy between the immunocytochemistry and western blotting results for NR2E3 in *erd* is difficult to reconcile at this time. As the results obtained in control retinas are comparable to what has been reported for other mammalian retinas, e.g. mouse and Nile rat [30], and monkey [31], we posit that the antibody is detecting NR2E3, and not an unrelated antigen. Lack of rod labeling in the mutant retina would suggest that the NR2E3 epitope is masked by the expression of the mutant STK38L protein. However, the mechanism for this finding is unknown, and we plan to address this question experimentally with GST pull down assays to examine for binding partners of mutant STK38L.

Further evidence of this change in the photoreceptor population from rods to hybrid rod/S-cones comes from the rod OS renewal and opsin biosynthesis studies. A fundamental difference between rods and cones is in the renewal of OS disc proteins [32]. Rod disc membranes are isolated, and autoradiograms following pulse labeling of newly formed discs with radiolabeled precursors show a distinct band of radioactivity that is displaced distally until shed from the OS tip. In contrast, cone OS disc membranes are not isolated, but continuous, and redistribution of labeled proteins by lateral diffusion results in a diffuse pattern of labeling [32], [33]. In *erd*, renewal was diffuse, and, unlike normals, a distinct labeled band could not be identified at any time point. Similar results could occur if OS morphogenesis is decreased, but only if the process is asynchronous as a uniform decrease would still result in renewal by band displacement, albeit at a slower rate [34].

The complementary biochemical studies support the interpretation that newly synthesized radiolabeled opsin redistributes rapidly throughout the mutant OS, and loss of label occurs associated with the daily shedding of discs from the distal tips of the modified OS [32], [35]. This conclusion is based on finding comparable incorporation of both radiolabeled precursors into mutant and control rod opsin initially, but labeling decreased in mutants unlike normals. Decreased OS morphogenesis would result in a lower incorporation of label at the 2 and 4 day post injection time points. However, this lower rate of incorporation still would result in increased labeling over time, and not the decrease found in our study.

Thus the change from rod to hybrid rod/S-cone is associated with a change in rod OS structure where the membranous discs now become continuous with the plasma membrane. This would require not only a change in expression of the membrane associated proteins such as rod opsin and S-opsin, but also expression of a new repertoire of genes and proteins that determine photoreceptor OS structure. One such protein, RDS, is involved in cone OS biogenesis and maintenance, and appears to require formation of covalently linked RDS dimers to carry out this function [36]. Although RDS levels were only modestly elevated at 6.4 wks, distinct dimer formation was evident in the western blots.

Our results indicate that in the *STK38L* mutant retina terminally differentiated photoreceptors undergo cell division and differentiate into hybrid rod/S-cone photoreceptors. Because of the paucity of cones in the canine retina, and the finding that most of the proliferating cells are rods, we assume that reconstitution of the photoreceptor layer is rod-derived, although a cone contribution can not be excluded. The newly generated cells return to either the pool of rod or cone precursors, or upstream to the pool of undifferentiated postmitotic photoreceptor cells [37]. Although we have no direct experimental data to support either alternative, the low levels of CRX (western blots), and absent NR2E3 photoreceptor layer labeling at the time when a new population of photoreceptors is being generated argues for the newly produced cells to come from the rod and possibly the cone precursor pool. However, a cautious interpretation of these findings is important given that expression changes in the *erd* retina, which is terminally differentiated yet photoreceptors are dying or dividing, may have limited similarities to what happens in normal retinal development with expression of transcription factor genes involved in photoreceptor cell specification [17].

In summary, the *STK38L* mutant retina is atypical in that terminally differentiated photoreceptors undergo cell death or proliferation, and generate a new class of hybrid rod/S-cone photoreceptors. The results suggest a role for STK38L in the control of cell division and morphogenesis in photoreceptors and possibly other retinal neurons. Future studies will inform on the role of the N-terminal regulatory region in these functions, and suggest approaches that can be manipulated experimentally to reconstitute and preserve a diseased photoreceptor layer.

## Materials and Methods

### Animals

Dogs were maintained at the Retinal Disease Studies (RDS) facility in Kennett Square, PA, and supported by NEI/NIH (EY-06855) and Foundation Fighting Blindness Center grants. The study was carried out in strict accordance with the recommendations in the Guide for the Care and Use of Laboratory Animals of the National Institutes of Health, and adhered to the ARVO Resolution for the Use of Animals in Ophthalmic and Vision Research. The protocols were approved by the Institutional Animal Care and Use Committee of the University of Pennsylvania (IACUC Protocol #s 801870, 802467, 803269), and all efforts were made to minimize suffering. Intravitreal injections for outer segment renewal and opsin biosynthesis studies were made in dogs anesthetized with thiopental sodium; tissue collection was performed under deep pentobarbital anesthesia, and was followed by euthanasia with an overdose of the same or comparable (Euthanol) agent. The dogs represent an outbred population with a common genetic background segregating *erd* and other retinal disease alleles [1]. The genotypes, ages studied and procedures performed are detailed in Table 1.

**Table 1:** Summary of procedures and dogs used in the studies.

Studies	Disease Status	Dogs (or eyes)	Ages-wks (#/time point)	Procedures
Anatomy				
	Control*	11	Preterm, 1, 1.6, 4, 4.7, 7, 8.7, 9, 12.3 (2), 25.7	Paraformaldehyde fixation for immunohistochemistry, TUNEL, cell counting, structural assessment
	Mutant	13	4.3, 7.7 (2), 8.3, 8.4, 9.1 (2), 9.3, 10.4, 11.6 (2), 12.3, 14.1	
	Mutant	11	7.1 (3), 7.3, 9.1 (2), 48.1, 62, 68, 101, 165	Bouin's fixation for cell counting, structural assessment
Gene/Protein Expression				
	Control	11	3 (3), 7 (3), 7.4, 8.2, 16 (3)	qRT-PCR, western analysis
	Mutant	10	6.4 (3), 8.3, 9.6, 9.9 (2), 11.9(2), 14.1	
Outer Segment Renewal Ω				
	Control**	(4)	8.7, 10.9, 16.4 (2)	Light microscopic autoradiography with ^3^H-leucine (6 eyes) or ^3^H-fucose (6 eyes)
	Mutant	(8)	8.3, 8.7, 9 (3), 10.9	
Opsin Synthesis Ω				
	Control**	(9)	9.9 (2), 16.4 (4)	Dual labeling with ^3^H-fucose/^14^C-leucine, opsin isolation, quantification of radioactivity
	Mutant	(15)	8.3 (6). 9 (4)	

* -one control dog is *erd* heterozygous.

** -four control dogs are *erd* heterozygous.

Ω -fellow eyes from 5 dogs (2 control; 3 *erd* affected) were used in the outer segment renewal and opsin synthesis studies.

### Anatomic/immunochemical studies

For structural, cell counting, immunocytochemical, TUNEL and cell proliferation studies, eyecups were fixed in 4% paraformaldehyde (PF), and central superior and inferior retinal strips (optic disc to ora serrata) were embedded in optimal cutting temperature medium (OCT; Sakura Fientek, Torrance, CA) using standard methods [38]. Archival tissues fixed in Bouin's solution and stored in 70% ethanol from affected animals were used for structural and cell counting studies. Sections from paraffin embedded tissues were cut in the dorso-ventral plane through the pupil-optic disc axis.

Sections from both the superior and inferior meridians were examined in contiguous fields from the optic disc to the ora serrata; this included evaluation of the rod and cone IS and OS, and the thickness of the ONL. For each dog, a section from the superior and inferior quadrants was used for quantitative evaluation of the ONL cell counts at two specific locations: A1 = 2000±500 µm from the optic nerve edge, and A2 = midpoint (equidistant from optic disc and ora serrata) of the retina ±500 µm. At each of these sites, the number of rows of nuclei in the ONL were counted in three areas of a 40× field and averaged.

Fluorescent immunohistochemistry was done on 7 µm cryosections taken from the superior and inferior retinal meridians, and incubated overnight at 4°C or at room temperature for 1 hour with the primary antibodies after a blocking step with 1.5% BSA/PBS, 0.25% Triton X-100 (Sigma-Aldrich, St. Louis, MO). The primary antibodies and cell markers used, cell class specificity, and target protein are detailed in Table S1, and include: *cones*: human cone arrestin, PNA, CNGA3, L/M- (COS-1) or S- (OS-2) cone opsins [39], red/green cone opsin, blue cone opsin; *rods*: rod opsin; *Müller cells, microglia and macrophages*: PAX6, GS, CD18; *cell proliferation, retinal stem cells*: PCNA, KI-67, PHH3, Nestin; *transcription factors*: PAX6, NR2E3, NRL. Apoptotic nuclei were visualized by TUNEL (terminal deoxynucleotidyl transferase-mediated biotinylated UTP nick end labeling) with the *In Situ* Cell Death Detection kit (Roche Applied Science, Indianapolis, IN). Antigen retrieval was performed prior to PCNA and PHH3 labeling by heating in the presence of Antigen Unmasking Solution, High pH (Vector Laboratories, Burlingame, CA) using a microwave oven at 10% power.

Both single and double immunolabeling was used. The primary antibody pairs used for double immunolabeling were combinations of rabbit or goat polyclonal and mouse monoclonal antibodies. The antigen–antibody complexes were visualized with fluorochrome-labeled secondary antibodies (Alexa Fluor, 1∶200; Invitrogen, Carlsbad, CA), and 4′,6′-diamino-2-phenylindole (DAPI) stain was used to label cell nuclei. Slides were mounted with a medium composed of polyvinyl alcohol and DABCO (1,4 diazobizyklo-[2.2.2]oktan) (Gelvatol; Sigma-Aldrich), and examined with an epifluorescence microscope (Axioplan; Carl Zeiss Meditec, Thornwood, NY). Epifluorescence or transmitted light images were captured with a Spot 4.0 camera (Diagnostic Instruments, Inc., Sterling Heights, MI) and imported into a graphics program (Photoshop and Illustrator; Adobe, San Jose, CA) for display. When precise localization of markers was needed, sections were also imaged by confocal microscopy using a Nikon A1R Laser Scanning Confocal Microscope with DUS 32 Spectral detector (Nikon Instruments, Melville, NY) through a 63× Plan APO objective lens with 1.2 numerical aperture. The specimens were excited at 488 and 561 nm, respectively, with multi line Argon and DPSS lasers.

TUNEL-, PCNA- or PHH3-labeled cells in the ONL were counted throughout the entire length of the section. In determining the proportion of photoreceptor cells that undergo cell death or proliferation as a function of time, the results were expressed as the number of TUNEL, PCNA or PHH3 labeled photoreceptor cells per 10^6^ µm^2^ of ONL [11]. The area of the ONL of each section was obtained by measuring the entire length of the ONL from the optic disc to ora serrata, and multiplying it by the average thickness of the ONL throughout the section (mean value of the thickness measured in ten evenly distributed locations). For each retina examined for TUNEL, PCNA or PHH3 labeling the procedure was performed in triplicate with sequential sections taken from both the superior and inferior meridians. The values were averaged and reported as the mean ±1 SD.

We determined qualitative differences in immunolabeling intensity between normal and mutant retinas with antibodies directed against S- and L/M-cones. For this, sections (2) from selected age groups were incubated with different dilutions of the antibodies to determine the lowest concentration needed to provide comparable results to those obtained with the routine working concentrations (Table S1). These results were validated in repeat analyses.

### Gene expression

Quantitative real time-PCR (qRT-PCR) was used to assess expression of selected genes at different time points of normal development (3 (n = 3) and 7 (n = 3) wks) and disease (6.4 (n = 2), 8.3/9.9 (n = 3) and 11.9/14.1 (n = 2) wks). Retinas from 16 wk (n = 3) normal dogs served as reference control. Total RNA was isolated by Trizol extraction (Invitrogen-Life Technologies, Carlsbad, CA), and concentration measured with a Nanodrop 1000 spectrophotometer (Thermo Fisher Scientific, Wilmington, DE). RNA samples were treated with RNase-free DNase (Ambion, Austin, Tx), and two µg total RNA from each sample was used for cDNA synthesis using the High Capacity cDNA reverse transcriptase Kit (Applied Biosystems (ABI), Foster City, CA). Quantitative RT-PCR was performed on a 7500 Real Time PCR System and software v2.0 (ABI) using 30 ng cDNA from each sample, and amplified using Taqman assays with canine-specific ABI inventoried probes for *STK38L/NRD2*, *LATS1* (Cf02626754_m1) and *CCNA1* (Cf02633425_m1); SYBR green analysis was used for *CCND1* (Forward: CATCTACACTGACAACTCCATCC; Reverse: CAGGTTCCACTTCAGTTTGTTC). For analysis of *STK38L/NDR2* one probe was located within the exonic deletion (*STK38L (exon 4*) = Cf02709228_m1), and the second in exon 6, 3′ to the exon 4 splicing defect, and used to exclude alterations in splicing resulting from the SINE element insertion (*STK38L (exon 6*) = Cf02634613_m1). CT values of each gene were normali zed to *GAPDH*, and comparisons between groups were done with the ΔΔCT method [40]. Statistical significance between different groups in comparisons to the 16 wks old normal control retinas was assessed with an unpaired *t*-test, and expressed as statistically significant (p≤0.05) or towards statistical significant (0.05≤p≤0.1).

### Immunoblotting

Equal amounts of total protein as determined by BCA Protein Assay Kit (former Pierce Biotechnology now Thermo Fisher Scientific, Rockford, IL) were separated by 10% SDS-PAGE under reducing conditions, immunoblotted and probed with antibodies. These are detailed in Table S1 and include antibodies against red/green opsin, blue opsin, CNGA3, NRL, NR2E3, RDS/peripherin, and CRX. Appropriate HRP-conjugated secondary antibodies were used subsequently, and an antibody against β-actin was used as a loading control. Densitometric analysis of the blots was performed on Kodak Image Station 4000MM (Molecular Imaging Systems, Carestream Health, Rochester, NY). The net intensity was corrected for background intensity observed. Values in figure are expressed as corrected net intensity of the sample normalized to corrected net intensity of actin loading controls. Densitometry data were analyzed using Sigma Stat Version 3.1, and intensities from four independent measurements on two westerns were analyzed by Student t test using 95% or 99% confidence intervals.

### Rod outer segment renewal and opsin biosynthesis

Rod OS renewal and opsin biosynthesis were examined at specific time points following the injection of ^3^H-fucose or ^3^H-leucine (OS renewal) or a combination of ^3^H-fucose/^14^C-leucine (opsin biosynthesis) into the vitreous of anesthetized dogs using previously described methods [34]. Post-injection time points were 1, 2, 3 and 4 days for OS renewal, and 1, 2 and 4 days for opsin biosynthesis (Table 1). In the 4 day interval following injection, the rods have renewed ∼50% of their OS due to continuous addition of new discs at the base [34]. For the renewal studies, the eyes were fixed in mixed-aldehyde/osmium solution, embedded in plastic resin, and 1 µm thick sections were coated with a photographic emulsion and maintained at 4°C in the dark until developed [34]. Two series of experiments were conducted to examine opsin biosynthesis, and loss of opsin labeling over time after injection. For these studies, eyes received a combination of ^3^H-fucose and ^14^C-leucine intravitreally. Eyes from anesthetized dark adapted dogs were enucleated under dim red light, and crude rod outer segment preparations were made by vortexing and centrifugation in 40% (w/v) sucrose. After sonication, detergent solubilized rod OS proteins were separated in a 10% polyacrylamide gel, stained with Coomassie blue, and gel slices digested and counted in a scintillation counter [34]. Disintegrations per minute (DPM) in the opsin peaks were normalized to the highest protein value for each set of gels, and to the DPM count for the post injection day 1 control eye of the same series.

## Supporting Information

Figure S1 **Phospho-histone H3 (PHH3, red) and glutamine synthetase (GS, green) double labeling of** ***erd*** **mutant retinas.** *(* ***A1, B1*** *)* Merged images of retinas at 7.7 (A) and 11.6 (B) weeks of age show PHH3 labeled nuclei only in ONL (arrows); *(A2, B2)* the images of GS labeling shows the lack of label in spaces (arrows) occupied by the PHH3 labeled nuclei. Arrowheads in A1, A2 point to intensely labeled end feet of Müller cells; * = large retinal ganglion cell surrounded by GS positive processes. Scale bar = 20 µm; DAPI (blue) nuclear staining.
(TIF)

Figure S2 **Nestin, cd18 and PAX6 labeling.** Representative single (A1,2-Nestin, B1,2-CD18; green) or double (C1,2-PAX6-red, rod opsin-green) labeling, with DAPI (blue) nuclear staining of normal or *erd* retinas of different ages. *(A1, A2)* Nestin labels cells only in the retinal periphery (arrowhead) near the ora serrata (ora). *(B1, B2)* CD18 labeling of microglia is limited to the outer and inner plexiform layers (*). *(C1, C2)* A population of cells in inner border of the INL, presumably Müller cells, labels intensely with PAX6 (arrowheads); labeling is also present in the ganglion cell layer (arrows). Note rod opsin delocalization into the mutant ONL (C2). Scale bar = 20 µm.
(TIF)

Table S1 Antibodies and reagents used for immunohistochemistry and immunoblotting.
(DOC)
