# Supplementary figures and images for: Photoreceptor Cell Death, Proliferation and Formation of Hybrid Rod/S-Cone Photoreceptors in the Degenerating STK38L Mutant Retina
Source: PLoS One. 2011 Sep 30;6(9):e24074. doi: 10.1371/journal.pone.0024074 (PMC3184085; doi:10.1371/journal.pone.0024074)

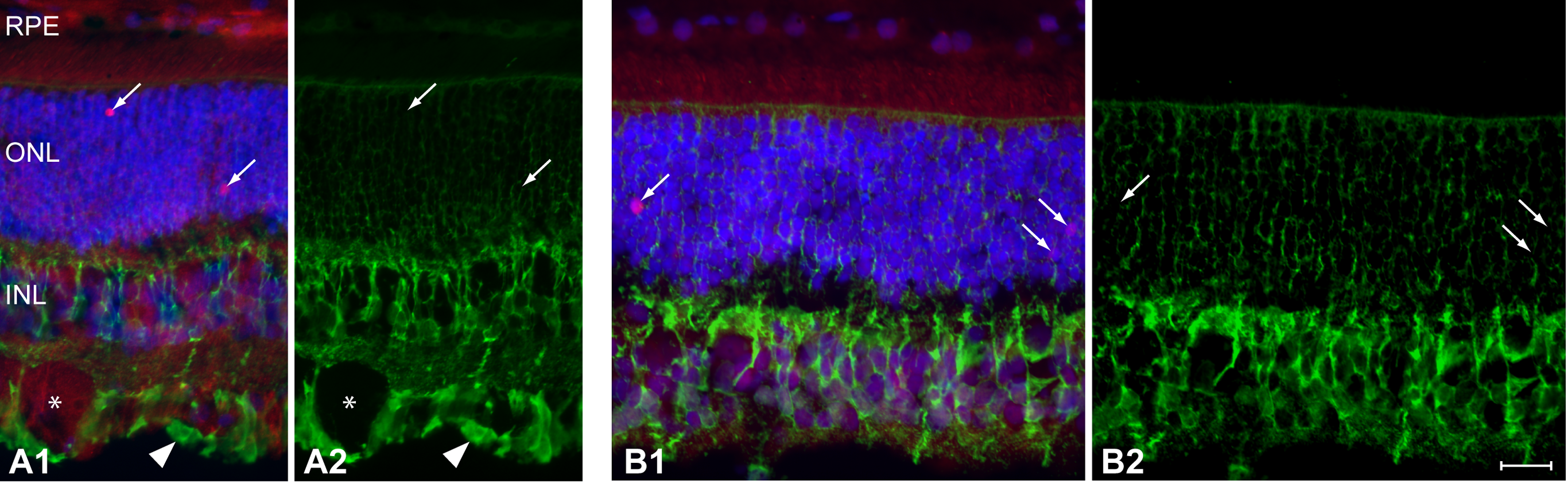

Supplement: Figure S1 — Phospho-histone H3 (PHH3, red) and glutamine synthetase (GS, green) double labeling of erd mutant retinas. ( A1, B1 ) Merged images of retinas at 7.7 (A) and 11.6 (B) weeks of age show PHH3 labeled nuclei only in ONL (arrows); (A2, B2) the images of GS labeling shows the lack of label in spaces (arrows) occupied by the PHH3 labeled nuclei. Arrowheads in A1, A2 point to intensely labeled end feet of Müller cells; * = large retinal ganglion cell surrounded by GS positive processes. Scale bar = 20 µm; DAPI (blue) nuclear staining. (TIF) [file pone.0024074.s001.tif]

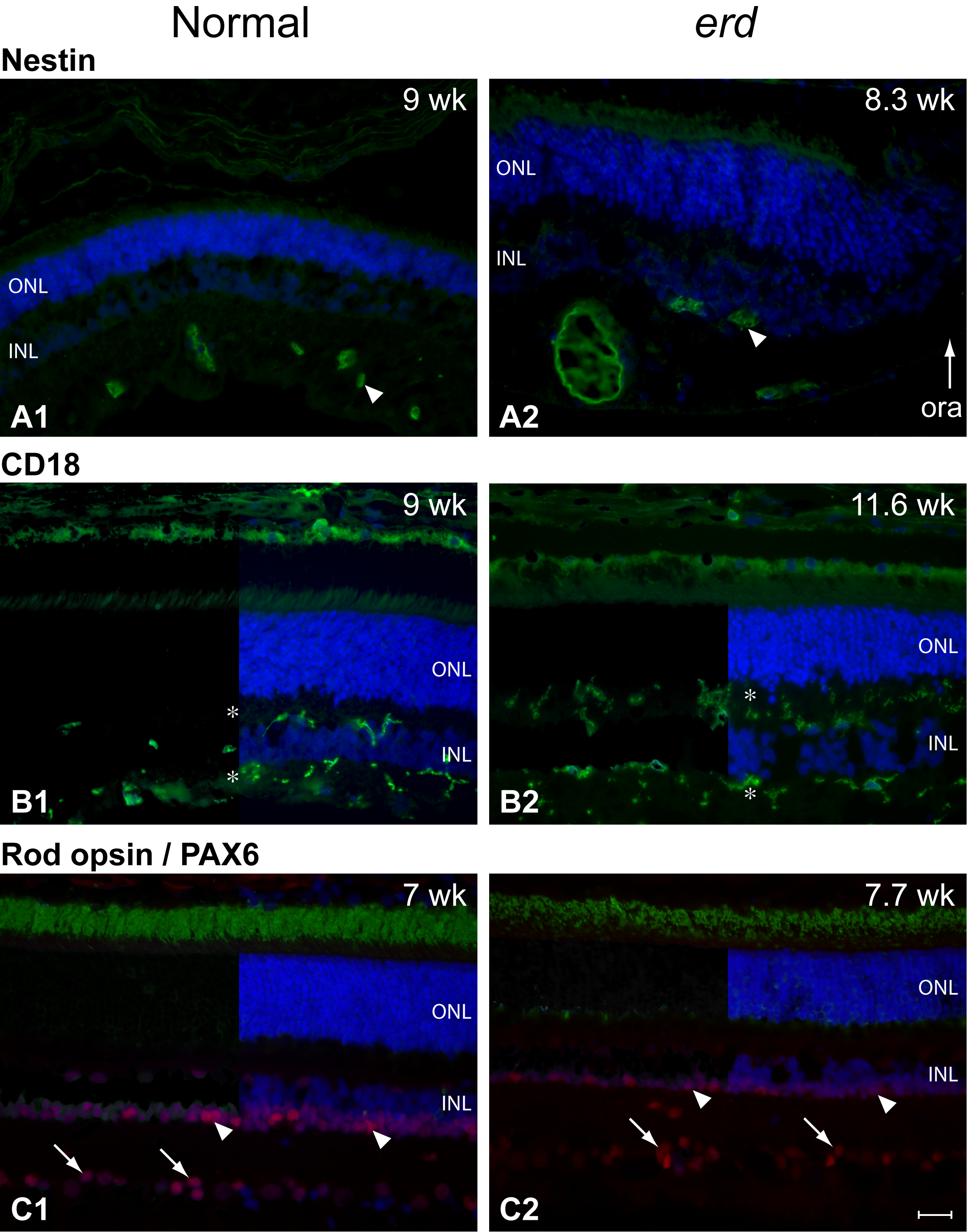

Supplement: Figure S2 — Nestin, cd18 and PAX6 labeling. Representative single (A1,2-Nestin, B1,2-CD18; green) or double (C1,2-PAX6-red, rod opsin-green) labeling, with DAPI (blue) nuclear staining of normal or erd retinas of different ages. (A1, A2) Nestin labels cells only in the retinal periphery (arrowhead) near the ora serrata (ora). (B1, B2) CD18 labeling of microglia is limited to the outer and inner plexiform layers (*). (C1, C2) A population of cells in inner border of the INL, presumably Müller cells, labels intensely with PAX6 (arrowheads); labeling is also present in the ganglion cell layer (arrows). Note rod opsin delocalization into the mutant ONL (C2). Scale bar = 20 µm. (TIF) [file pone.0024074.s002.tif]
