# Supplementary material for: Photoreceptor Cell Death, Proliferation and Formation of Hybrid Rod/S-Cone Photoreceptors in the Degenerating STK38L Mutant Retina
Source: PLoS One. 2011 Sep 30;6(9):e24074. doi: 10.1371/journal.pone.0024074 (PMC3184085; doi:10.1371/journal.pone.0024074)
Supplement: Table S1 — Antibodies and reagents used for immunohistochemistry and immunoblotting. (DOC) [file pone.0024074.s003.doc]

Table S1. Antibodies and reagents used for immunohistochemistry and immunoblotting.

| **Markers** | **Host** | **Normal retinal localization or target protein** | **Working dilution** | **Source, Catalog No. or Name#** |
| --- | --- | --- | --- | --- |
| **Immunohistochemistry** |  |  |  |  |
| **Cone** |  |  |  |  |
| Human cone arrestin  (hCAR) | rabbit polyclonal | cones | 1:10000 | C. Craft |
| PNA-rhodamine lectin | lectin | cone insoluble matrix | 20 µg/ml | Vector Laboratories, RL-1072 |
| CNGA3 | rabbit polyclonal | cone OS | 1:5000 | A. Komáromy |
| COS-1 | mouse monoclonal IgG3 | L/M-cone OS | 1:100* | Á. Szél |
| Red/green opsin | goat polyclonal | L/M-cone OS | 1:100* | SCBT, sc-22117 |
| Red/green opsin | rabbit polyclonal | L/M-cone OS | 1:5000* | Millipore, AB5405 |
| OS-2 | mouse monoclonal IgG3 | S-cone OS | 1:100* | Á. Szél |
| Blue opsin | goat polyclonal | S-cone OS | 1:100* | SCBT, sc-14363 |
| Blue opsin | rabbit polyclonal | S-cone OS | 1:5000* | Millipore, AB5407 |
|  |  |  |  |  |
| **Rod** |  |  |  |  |
| Rhodopsin | mouse monoclonal IgG1 | rod OS | 1:1000 | Millipore, MAB5356 |
| Rhodopsin | mouse monoclonal IgG1 | rod OS | 1:1000 | Millipore, MAB5316 |
| Rhodopsin | rabbit polyclonal | rod OS | 1:100 | SCBT, sc-15382 |
|  |  |  |  |  |
| **Müller cells and microglia** | |  |  |  |
| Glutamine Synthetase | mouse monoclonal IgG2b | Müller cells | 1:20,000 | Chemicon, MAB302 |
| CD18 (CA16:3C10) | mouse monoclonal IgG1 | canine monocytes, granulocytes, microglia | 1:10 | P. Moore |
|  |  |  |  |  |
| **Cell proliferation or cell death** | |  |  |  |
| PCNA | mouse monoclonal IgG2a | proliferating cell nuclear antigen | 1:100 | Millipore, MAB424 |
| KI67 | monoclonal mouse IgG1 | nuclear cell proliferation antigen | 1:20 | BD Pharmingen, 556027 |
| Phospho Histone H3 (Ser 10) | rabbit polyclonal | cell mitosis | 1:50 | Millipore, 06-570 |
| Phospho Histone H3 (Ser 10)(6G3) | mouse monoclonal | cell mitosis | 1:50 | Cell Signaling Tech, 9706 |
| TUNEL In Situ Cell Death Detection Kit, Fluorescein |  | apoptotic cell nuclei | kit instructions | Roche, 11684 795 910 |
|  |  |  |  |  |
| **Stem cells** |  |  |  |  |
| Nestin, clone 10C2 | mouse monoclonal IgG1 | neuronal stem cells | 1:100 | Millipore, MAB5326 |
|  |  |  |  |  |
| **Transcription factors** |  |  |  |  |
| NR2E3  NRL  PAX6 | rabbit polyclonal  rabbit polyclonal  rabbit polyclonal | Nr2e3 (human)  Nrl (human)  INL | 1:100  1:100  1:1000 | A. Swaroop  A. Swaroop  Covance, PRB-278P |
|  |  |  |  |  |
| **Immunoblotting** |  |  |  |  |
| Red/green opsin | rabbit polyclonal | L/M-cone OS | 1:300 | Abcam, ab65695 |
| Blue opsin | rabbit polyclonal | S-cone OS | 1:5000 | Millipore, AB5407 |
| CNGA3 | rabbit polyclonal | cone OS | 1:5000 | A. Komáromy |
| NRL (H-120) | rabbit polyclonal | Nrl | 1:200 | SCBT, sc-33183 |
| NR2E3 (N-13) | goat polyclonal | Nr2e3 | 1:200 | SCBT, sc-46209 |
| RDS/peripherin 3B6 | mouse monoclonal IgG | rod and cone OS | 1:1000 | R. Molday |
| CRX | rabbit polyclonal | Crx | 1:200 | SCBT, sc-30150 |
| Actin | rabbit polyclonal | loading control | 1:500 | SCBT, sc-10731 |

Key: * lower concentrations of these antibodies were used to determine relative expression. See Materials and Methods.

**#**=Commercial sources for antibodies: Abcam, Cambridge, MA, BD Pharmingen, San Diego, CA, Cell Signaling, Danvers, MA, Covance, Princeton, NJ, DAKO North America, Carpinteria, CA, Millipore, Billerica, MA, Roche Applied Science, Indianapolis, IN, Santa Cruz Biotechnology, Inc. Santa Cruz, CA, Vector Laboratories, Inc., Burlingame, CA
